# Association between COVID-19 and Sick Leave for Healthcare Workers in a Large Academic Hospital in Southern Italy: An Observational Study

**DOI:** 10.3390/ijerph19159670

**Published:** 2022-08-05

**Authors:** Raffaele Palladino, Michelangelo Mercogliano, Claudio Fiorilla, Alessandro Frangiosa, Sabrina Iodice, Stefano Sanduzzi Zamparelli, Emma Montella, Maria Triassi, Alessandro Sanduzzi Zamparelli

**Affiliations:** 1 Department of Public Health, University of Naples “Federico II”, 80125 Naples, Italy; 2 Department of Primary Care and Public Health, Imperial College London, London W6 8RP, UK; 3 Interdepartmental Center for Research in Healthcare Management and Innovation in Healthcare (CIRMIS), University of Naples “Federico II”, 80131 Naples, Italy; 4 Division of pneumology, A. Cardarelli Hospital, 80131 Naples, Italy; 5 Department of Clinical Medicine and Surgery, Section of Respiratory Diseases, University “Federico II”, Azienda Ospedaliera dei Colli-Monaldi Hospital, 80131 Naples, Italy

**Keywords:** COVID-19, SARS-CoV-2, sick leave, symptoms, public health, healthcare workers, CAT score

## Abstract

Studies have shown that the pandemic has led to an increase in sick leave periods among healthcare workers (HCWs); however, this might have changed over time considering increase in vaccination coverage and change in COVID-19 variant predominance. Therefore, we conducted an observational study to evaluate whether the type of symptoms and the duration of sick leave period for healthcare workers working in a large university hospital in the South of Italy changed between January 2021 and January 2022; 398 cases of COVID-19 were identified for a total of 382 subjects involved. A total of 191 subjects answered the questionnaire about symptoms; of these, 79 had COVID-19 during the period from March 2020 until February 2022. The results showed a decrease of about 1.2 days in sick leave period for each quarter without finding significant differences in the perception of symptoms. It is possible to hypothesize a contribution from the Omicron variant to the decrease in sick leave period in the last quarter, from vaccination coverage, from optimization of COVID-19 management, and from change in the regulations for the assessment of positivity.

## 1. Introduction

From 2019 to today, coronavirus disease (COVID-19) has massively impacted health systems worldwide, leading to a significant increase in morbidity and mortality [1], due to its high transmissibility, high contagiousness [2], and the emergence of new variants of SARS-CoV-2, such as the Alpha (B.1.1.7 lineage), Delta (B.1.617.2 lineage), and Omicron (B.1.1.529 lineage); the latter two lineages are currently variants of concern (VOC) [3].

All these variants have been or still are responsible for waves of infections around the world: Alpha variant (B.1.1.7 lineage) was first detected in the UK in September 2020 [4], and was the dominant variant in Italy until July 2021 [5]; Delta variant was originally found in India in December 2020 [6] and was the dominant variant in Italy from June 2021 to December 2021, being replaced by the Omicron variant from January 2022 [7]. The Omicron variant was first reported in South Africa in November 2021 and has the highest number of mutations among the VOCs, which gives it greater transmissibility [2], immune evasion [8], and greater resistance to neutralizing antibodies [9] and vaccine-induced humoral immunity [10]. However, studies have shown that the Omicron variant may have poorer replication in the lungs when compared with the Delta variant [11] and attenuated replication in lower respiratory tissues [12]. This change in tropism could potentially lead to increased transmissibility [13], but there has been reported a reduced risk of severe COVID-19 in individuals who are infected with Omicron compared to those infected with Delta variant [14], which might also translate into a reduced sick leave period for those affected.

The pandemic has significantly affected working capacity, especially for healthcare workers (HCWs), but scientific evidence is still sparse [15,16,17], especially regarding differences in relation to the genetic evolution of the virus and the consequent variation in symptoms, and whether this might have translated into differences in the COVID-19 burden on working capacity over time.

Studies have shown that the pandemic has led to an increase in sick leave periods among the HCWs [15,16], that vaccinated HCWs have a reduced incidence and a shortened sick leave length than before vaccination [18], that return-to-work time for fully vaccinated HCWs was significantly shorter than that of partially vaccinated/unvaccinated HCWs, that fully vaccinated HCWs also showed milder symptom profiles compared to partially vaccinated and unvaccinated HCWs [19], and that sick leave utilization among HCWs declined significantly after vaccines became available [20].

Increase in vaccination coverage, symptoms evolution for different VOCs, as well as improvements in clinical management of the COVID-19 infection might have all played a large role on the impact of sick leave due to COVID-19 and return-to-work timing for HCWs; however, evidence is still limited. Therefore, we conducted an observational research study combining a retrospective and cross-sectional study to evaluate whether the type of symptoms and the duration of sick leave period for HCWs working in a large university hospital in the South of Italy changed between January 2021 and January 2022.

## 2. Materials and Methods

### 2.1. Study Design

Considering the change variants of SARS-CoV-2 over time, the change in the methods for assessing positivity for the virus, and the improvement in the efficiency of the return process following sick leave, the relationship between the chronological period in which the HCWs contracted the SARS-CoV-2 infection and the duration of the sick leave was evaluated.

We conducted a retrospective study employing a time series analysis which used routinely collected administrative data regarding HCWs sick leave period due to COVID-19 between January 2021 and January 2022 extracted from the “Federico II” Hospital, a large university hospital in the Campania Region of Italy. Furthermore, we conducted a cross-sectional survey in March 2022 targeting HCWs working at the University Hospital to assess severity of COVID-19 symptoms for those who tested positive between March 2020 and February 2022. The survey was based on an anonymized questionnaire including the modified CAT score; subjects self-reported symptoms they presented during COVID-19 positivity. Data were completely anonymized to be processed safely. The study obtained ethical approval from the “Federico II University” Ethics Committee (prot. 65/21).

### 2.2. Study Variables

Sick leave period was extracted from the University Hospital data warehouse and expressed in days. Severity and typology of COVID-19 symptoms were evaluated using the modified COPD Assessment Test (CAT), which is a scoring system, containing eight items, for COPD patients and assesses the impact on health [21,22]. The CAT was also used in assessing the impact of COVID-19 [23,24,25]. The modified CAT is a specific test for assessing the impact of COVID-19; it consists of nine items, as the pyrexia assessment has been added.

Main variable of interest was time expressed in trimester due to sample size constraints. Additional study variables included: age, sex, vaccination status, booster dose, and COVID-19 infection after vaccination.

### 2.3. Statistical Analyses

Association between time and sick leave duration was evaluated by employing mixed-effect linear regression models, controlled for age, sex, and vaccination status. To account for the possibility that HCWs might become infected twice, person was included in the model as random intercept. Change in severity of COVID-19 symptoms over time was evaluated by employing multivariable linear regression models controlled for age, sex, booster dose, and COVID-19 infection after vaccination.

## 3. Results

**Figure 1:**
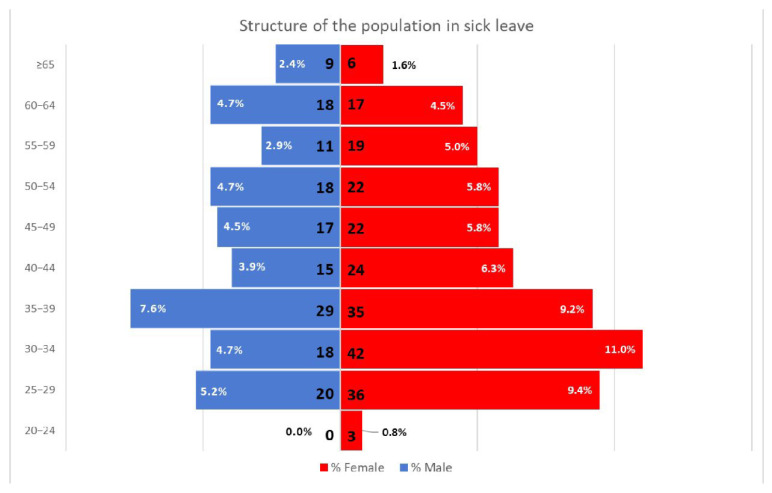
**The population of subjects who had sick leave during the study period**.

As of January 2022, 2083 HCWs (54% female) were employed at the University Hospital “Federico II” of Naples. In total, 398 cases of COVID-19 were identified, for a total of 382 subjects involved (about 18.3% of HCWs; average age: 42.74), of which 227 were women (average age: 41.48) and 155 were males (average age: 44.59) (Figure 1). As of January 2022, 89 people were vaccinated with two doses (23.3% of population; 51 females and 38 males), 282 with also the booster dose (73.8% of population; 172 females and 110 males), and only 11 people were not vaccinated (2.9% of population; 4 females and 7 males). Our analysis also considered the time of infection in relation to vaccination and found that 179 people had contracted the virus before vaccination (46.8% of population; 109 females and 70 males), 73 after the primary course of vaccination (19.1% of population; 41 females and 53 males), and 130 after the booster dose (34% of population; 77 females and 53 males).

**Figure 2:**
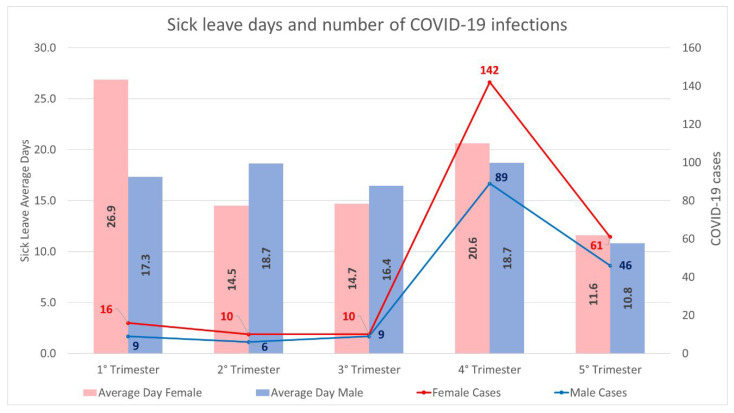
**Distribution of COVID-19 cases over the study period.** The graph represents the number of COVID-19 cases divided between males and females on a quarterly basis; the average days of sick leave by gender are also shown. The database includes 13 months; the 5th trimester consists of January 2022 only.

The sick leave cases are distributed temporally as shown in Figure 2; the cases are distributed over the quarters and the average duration, in days, of the sick leave is shown.

As shown in Table 1, controlling for age, sex, and vaccination status in relation to sick leave period, the linear mixed model estimated that between January 2021 and January 2022, on average, for each trimester, a reduction of 1.2 days in the average sick leave period was found (coef. −1.23; 95% CI: −2.39/−0.06).

**Figure 3:**
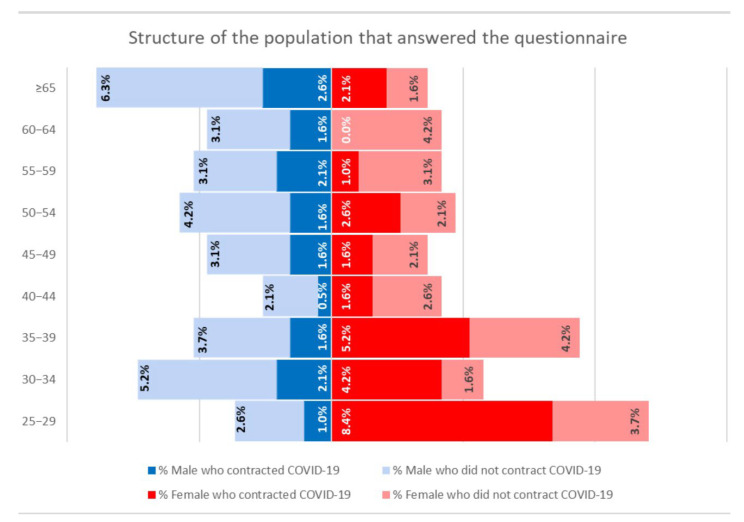
**Structure of the population that completed the questionnaire**. In total, 191 subjects answered the questionnaire; of these, 79 had COVID-19. Of the 99 women, 51 had COVID-19; of the 92 men, 28 had COVID-19. Only positive subjects also completed the modified CAT.

**Figure 4:**
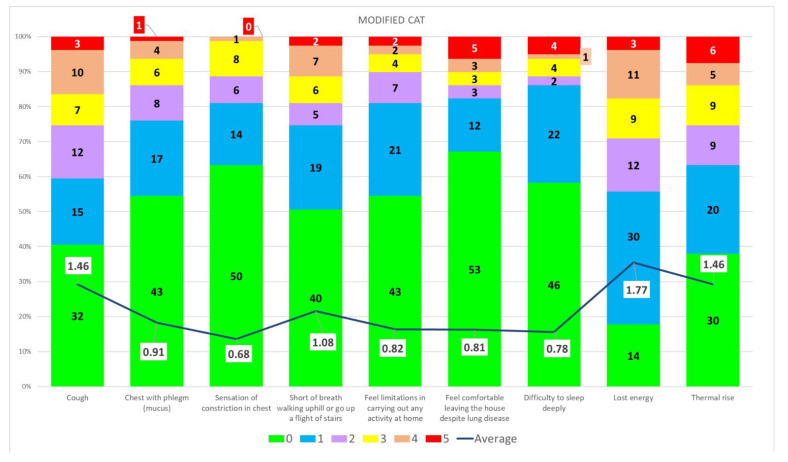
**Severity of the COVID-19 symptoms in the study population.** In completing the modified CAT, the patient must indicate the severity of symptoms from 0 to 5 based on the symptomatic examples shown in the modified CAT. The graph shows the numbers of cases, numerically, that have a certain score assigned to a certain symptom; 0 indicates that the subject did not have the symptom, 5 is the highest grade. The line represents the average score assigned to a type of score by the interviewees.

The questionnaire, including the modified CAT, was administered anonymously to the staff working at the University Hospital. In total, 191 responses were collected regarding vaccination status and whether or not the subjects contracted SARS-CoV-2 infection. Of these, 79 reported having contracted COVID-19 at least once; of these, 4 reported having contracted COVID-19 a second time. The structure of the population that responded to the questionnaire is shown in Figure 3. Regarding the first SARS-CoV-2 infection, the results of the collected responses are reported in Figure 4. The mean score of the modified CAT for the group under analysis was 9.77.

As shown in Table 2, the regression model found no difference in the mean score of the modified CAT in trimesters of the period from March 2020 until February 2022.

## 4. Discussion

This observational study assessed whether duration and symptoms of COVID-19 infection changed for HCWs in a large University Hospital in Italy between January 2021 and January 2022.

There were 398 cases of sick leave period for a total of 382 subjects involved (about 18.3% of HCWs; average age: 42.74), of which 227 were women and 155 were men. On average, we found a 1.2 days decrease of sick leave for each quarter after controlling for age, gender, and vaccination status. In total, 191 questionnaires were administered anonymously to assess the change over time in COVID-19 symptoms. Seventy-nine subjects who replied had also contracted the infection. Our analysis found no association between the degree of symptoms estimated by the modified CAT and trimester of infection. As the most prevalent COVID-19 variant in Italy changed over the study period (Delta from to, Omicron from to) this decrease might be partially explained by the differences in infection length for COVID-19. Other possible factors influencing the findings included increase in vaccination coverage, optimization of COVID-19 management (i.e., change of sick leave duration in the case of asymptomatic cases), change from a bureaucratic point of view, and change in the regulations for the assessment of positivity (i.e., use of lateral flow tests for the diagnosis as well).

Although the population in the two analyses was heterogeneous considering occupation, age, and sex, it was homogenous for the vaccination status, due to the vaccination obligation for HCWs according to the Italian legislation and as reflected in our findings. Another study evaluated the duration of the sick leave period in a large Swedish population. The sick leave period was longer, with a duration of about 35 days, but the study is not comparable to ours due to main differences in study population (general population, not only HCWs) and because those who contracted the infection received sickness benefit during the period from 1 March to 31 August 2020 [26]. A further study, in Northern Italy, focused on HCWs; in addition to a decrease in incidence following vaccination, the median sick leave period was 12 days for unvaccinated HCWs and 11 days for vaccinated HCWs [18]. Our study mainly focused on the evaluation of variations of sick leave period with chronological time; therefore, it was in correlation with vaccination times, COVID-19 variants, and changes in the bureaucratic management of cases.

We found a decrease in the average days of sick leave without, however, a decrease in symptoms. Symptom perception can be influenced by personal perceptions [27,28] and specific populations, e.g., HCWs might perceive symptoms differently from the general population. Most primary cycle vaccinations are concentrated in the first trimester and booster doses in the fourth and fifth trimesters. Furthermore, previous studies that evaluated the symptoms using the CAT found high variability of the results, which was often related to variants prior to Omicron and with a differently stratified population by vaccination [23].

To the best of our knowledge this is one of the first studies that specifically looked at whether implementation of COVID-19 clinical management and change in the most highly prevalent VOCs over time were associated with differences in type of symptoms and the duration of sick leave period for HCWs. We conducted our research on HCWs working in a large university hospital in the South of Italy. Hence, results might be generalized to similar healthcare settings in the country. However, several caveats merit discussion. First, duration of sick leave period is also influenced by legislation changes over the study period regarding duration of quarantine period [26], which, however, impacted our findings slightly as the reduction in the minimum quarantine period for positive cases was implemented in January 2022, the end of our study period. Second, the use of a subjective tool, such as the CAT (even if modified with the added hyperpyrexia criterion), to assess the intensity of the symptomatology could be misleading. As evidenced in previous literature, there is a weak correlation between duration of the disease and intensity of symptoms [15,16,17]. Third, this specific analysis was based on a relatively small sample. This may have influenced the result due to a possible selection bias, as only personnel more willing to share their experiences, despite the data having been totally anonymized, might have decided to participate.

## 5. Conclusions

In our study, we found a decrease in the average sick leave due to COVID-19 in a large cohort of HCWs at the University Hospital “Federico II” of Naples. It is possible to hypothesize a contribution from the Omicron variant, which is less symptomatic, due to the decrease in sick leave period in the last quarter. Other possible factors influencing the findings included increase in vaccination coverage, optimization of COVID-19 management (i.e., change of sick leave duration in the case of asymptomatic cases), and change, from a bureaucratic point of view, in the regulations for the assessment of positivity (i.e., use of lateral flow tests for the diagnosis as well). Further studies should investigate whether the post-COVID-19 impact on quality of work and working ability for HCWs have changed over time.

## Figures and Tables

**Table 1:** **Factors associated with sick-leave period due to COVID-19**. Estimates were obtained by employing the linear mixed model controlled for age, sex, and vaccination status in relation to sick leave period. There is an association between the sick leave period of HCWs and the trimester during the period January 2021–January 2022.

Variable	Coefficient	[95% Confidence Interval]	
Trimester	−1.23	−2.39	−0.06
Sex—Male	−2.18	−4.39	0.04
Age	0.05	−0.04	0.14
Vaccinated	−3.11	−4.44	−1.78

**Table 2:** **Association between CAT score and clinical and demographic characteristics**. Estimates were obtained by employing the linear mixed model controlled for age, sex, and vaccination status in relation to CAT score. There is no association between the modified CAT score period of HCWs and the trimester during the period March 2020–February 2022.

Variable	Coefficient	[95% Confidence Interval]	
Trimester	1.04	−0.93	3.01
Sex—Male	−3.65	−7.93	0.64
Age	0.05	−0.11	0.20
Received booster dose	1.22	−4.93	7.38
COVID-19 infection after vaccine	−4.96	−16.11	6.19

## Data Availability

The data are not publicly available due to restrictions with Ethics and privacy.
